# Insulin Conformation Changes in Hybrid Alginate–Gelatin Hydrogel Particles

**DOI:** 10.3390/molecules29061254

**Published:** 2024-03-12

**Authors:** Gulzhan Ye. Yerlan, Michael Shen, Bakyt B. Tyussyupova, Sagdat M. Tazhibayeva, Kuanyshbek Musabekov, Paul Takhistov

**Affiliations:** 1 Department of Analytical, Colloid Chemistry and Technology of Rare Elements, Faculty of Chemistry and Chemical Technology, Al-Farabi Kazakh National University, 71 Al-Farabi Ave., Almaty 050040, Kazakhstan; yerlan.gulzhan@gmail.com (G.Y.Y.); baimuratovna78@mail.ru (B.B.T.); tazhibayeva_s@mail.ru (S.M.T.); musabekov40@mail.ru (K.M.); 2 Department of Food Science, The State University of New Jersey, 65 Dudley Road, New Brunswick, NJ 08901, USA; mxs3@rutgers.edu; 3 Petroleum Engineering Institute, Kazakh-British Technical University, Tole bi Street, 59, Almaty 050040, Kazakhstan

**Keywords:** hydrogel particles, insulin, insulin fibrinogenesis, gelatin, alginate

## Abstract

There is a strong need to develop an insulin delivery system suitable for oral administration and preserving natural (α-helix) insulin conformation. In this work, we fabricated alginate–gelatin hydrogel beads for insulin encapsulation. Altering matrix composition and crosslinking agents has resulted in various surface morphologies and internal spatial organization. The structures of the insulin-loaded matrices were studied using optical and field emission electronic microscopy. We use FTIR spectroscopy to identify insulin conformation changes as affected by the hydrogel matrices. It was found that blended alginate–gelatin matrices demonstrate better encapsulation efficiency and stronger swelling resistance to a simulated gastric environment than sodium alginate beads crosslinked with the CaCl_2_. FTIR measurements reveal conformation changes in insulin. It is also confirmed that in the presence of gelatin, the process of insulin fibrinogenesis ceases due to intermolecular interaction with the gelatin. Performed molecular modeling shows that dipole–dipole interactions are the dominating mechanism that determines insulin behavior within the fabricated matrix.

## 1. Introduction

There is a growing problem with the epidemic of the illness diabetes. The number of diabetes cases is about 537 million adults in 2021. This number is predicted to rise to 643 million by 2030 and 783 million by 2045 [1].

Insulin is a vital hormone that regulates blood sugar levels in the human body. Without insulin, glucose cannot penetrate the cell membrane effectively, leading to elevated blood sugar levels and severe health complications. A proper insulin level prevents hyperglycemia and protects against diabetic ketoacidosis. Insufficient insulin production requires external insulin administration to maintain appropriate blood glucose control.

The most convenient way of drug administration is the oral pathway. Insulin, a polypeptide hormone, consists of polypeptide chains: A-chain (21 amino acids) and B-chain (30 amino acids) connected by disulfide bridges. It is susceptible to enzymatic degradation in the gastrointestinal tract, especially in the stomach, where digestive enzymes (pepsin and trypsin) can rapidly degrade its structural integrity.

When administered orally, therapeutic proteins must withstand acidic pH values, dense cellular compounds, and thick layers of mucus before reaching systemic bioavailability [2]. Insulin encapsulation into the excipient matrix can provide a protective barrier that shields insulin from the degrading effects of gastric enzymes and the stomach’s acidic environment. Physical complexation between natural polymers such as alginate, chitosan, gelatin, and dextran can be used to design drug delivery systems for oral insulin administration [3,4].

Polysaccharides are of particular interest as effective candidates for the delivery of therapeutic agents due to their key parameters, such as non-toxicity, low immunogenicity, biocompatibility, homogeneity, and biological activity [5]. Polysaccharide-based drug delivery systems can ensure many drugs’ proper release and targeting [6,7,8]. Encapsulation of active pharmaceutical ingredients in a polysaccharide matrix makes it possible to change biodistribution profiles, reduce side effects, achieve controlled release, and protect against environmental factors [9]. Alginate is an ionic polysaccharide that forms a crosslink when divalent cations such as Ca^2+^ are added. When uronate blocks in alginate interact with calcium ions, crosslinking occurs, forming a compound called the “egg box” model.

The availability of various chemical modifications of the gelatin functional groups makes it a promising candidate for drug delivery system development [10]. In the study from [11], a two-layer film was developed from a mixture of gelatin and alginate, superior to a matrix consisting only of alginate. The addition of gelatin improved the physical properties and dispersion of the active pharmaceutical ingredient (API). X-ray diffraction studies have shown that the addition of gelatin increases the crystallinity of the film, indicating the presence of polymer-drug crystalline microaggregates in the films. When studying the uniformity of the drug content, it was found that the addition of gelatin leads to the formation of films with a uniform distribution of the drug in the matrix. Without gelatin, the API was grouped and not dispersed in the alginate film, as evidenced by the results of the SEM. These results indicate the possibility of gelatin contributing to the homogeneous dispersion of hydrophobic preparations in alginate films due to the formation of crystalline macro aggregates. Crosslinking agents such as carbodiimide glutaraldehyde are used to improve the mechanical properties of gelatin [12]. While gelatin may be able to support and improve the dispersion of medicinal compounds such as insulin, there may be additional issues with the digestion of gelatin. Since gastric fluid would readily dissolve gelatin in the digestive tract, premature release of medicinal compounds would result in ineffective practical applications [13]. Chemical crosslinking with glutaraldehyde results in stable hydrogels that are suitable for drug delivery applications [14,15]. A protective coating can combat the early digestion of gelatin and alginate gels. Glutaraldehyde is one example of a widely used crosslinking agent due to its high efficiency in stabilizing collagen materials [16]. The aldehyde group (–CHO) in the structure of glutaraldehyde can react with many functional groups, such as –NH_2_, –COOH, and –OH., which, as a result, makes it possible to improve the mechanical strength of composites [17]. This overall strength would be beneficial in protecting the alginate–gelatin structure underneath.

Optimizing the matrix composition and crosslinking conditions and understanding the environmental factors influencing the hydrogel matrix are crucial for controlling insulin release. This research aims to investigate how the protein-polysaccharide encapsulation matrix influences fibril formation and associated conformational changes in insulin. We intend to identify the most favorable intermolecular environment for stabilizing insulin by varying the hydrogel blend composition and employing different crosslinking agents.

## 2. Results

### 2.1. Matrix Design for the Hybrid Delivery System

We chose two model biopolymeric matrices widely used for drug delivery applications: sodium alginate as the primary matrix forming gelling agent and co-former gelatin. Combining the ionotropic gelation and covalent crosslinking in various combinations allows us to design several delivery matrices.

**Figure 1:**
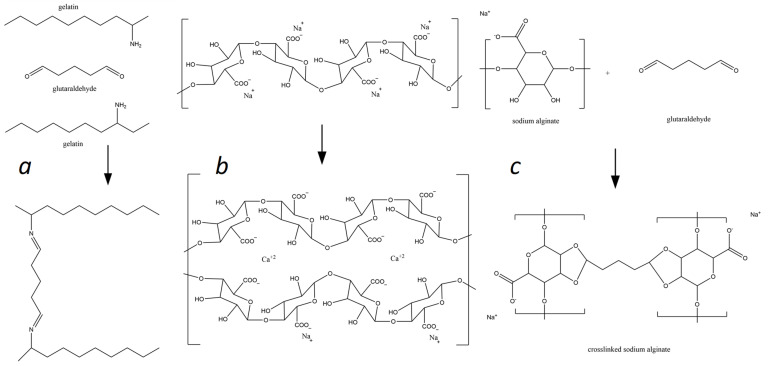
Mechanisms of the alginate and gelatin interactions with Ca^2+^ and GA ions: (**a**)—formation of the gelatin-glutaraldehyde complex; (**b**)—sodium alginate ionotropic gelation in presence of Ca^2+^ ions, and (**c**)—sodium alginate glutaraldehyde complexation.

Figure 1 shows the mechanisms of crosslinking of alginate and alginate–gelatin compositions in the presence of Ca^2+^ and GA ions. Carboxyl and polyhydroxy groups in alginate are highly reactive and as a result of the interaction of the Ca^2+^ ionic crosslinker with the G-blocks of the alginate chain, three-dimensional polymer networks are formed (see Figure 1b). Adding gelatin to sodium alginate should lead to electrostatic interactions between these biopolymers within the network. Glutaraldehyde can covalently interact with gelatin and alginate’s amino and hydroxyl groups as a chemical crosslinker, forming three-dimensional polymeric networks (see Figure 1a,c). It is expected that in the NaAlg-Gel (CaCl_2_-GA) matrix, all the mentioned interactions will occur since they contain both Ca^2+^ and GA crosslinkers and will contribute to strengthening the polymer network.

**Figure 2:**
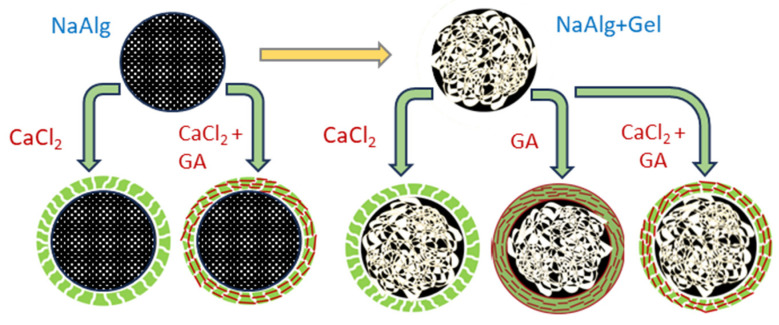
Design of the hydrogel matrices for insulin immobilization.

Based on suggested reaction mechanisms, we designed several hybrid systems with single and blended hydrogel compositions, using sodium alginate as a matrix-forming component and gelatin as the secondary gel-forming agent (see Figure 2). Combining two gel-forming substances with a mixture of two crosslinkers (CaCl_2_-GA) is expected to strengthen the polymer network since both electrostatic and covalent interactions will occur in such a three-dimensional polymer system.

### 2.2. Optimization of Gel Microbead Fabrication and Immobilization of Insulin into Polymer Matrices

**Figure 3:**
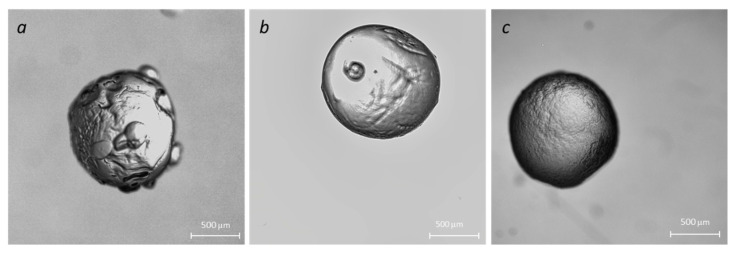
Effect of the ultrasound on the microparticle morphology: no ultrasound (**a**); ultrasound processing with 50% energy (**b**) and 100% energy (**c**).

Particles were fabricated using the external crosslinking method when droplets were injected from the syringe into a bath that contained a crosslinking agent [18]. Capillary needle diameter, material, and surface tension are the major factors that determine the size of the particles. When a biopolymer solution is pumped out of the needle, the droplet detaches from the capillary when the buoyancy force exceeds the surface tension at the droplet-needle interface. Droplet detachment is a complex process of jet breakdown due to the development of instabilities (initially Tollmien–Schlichting waves, then Raleigh instability) [1]. As the jet undergoes destabilization, tiny satellite droplets emerge from the primary droplet [19]. These satellite droplets are rapidly re-adhered to the surface of the “main” droplet, leading to complex and undesirable surface morphology (see Figure 3a). We used an ultrasound-assisted droplet generation to stabilize the droplet formation process [20]. A crosslinking bath was placed into a Symphony Ultrasonic Digital bath (VWR, Radnor, PA, USA) with operating frequency *f =* 35 KHz and variable amounts of ultrasound energy. Figure 3b,c depicts changes in the surface morphology of NaAlg + Cl_2_ beads fabricated at 50% and 100% of ultrasound energy, confirming the efficacy of the modified fabrication method.

**Figure 4:**
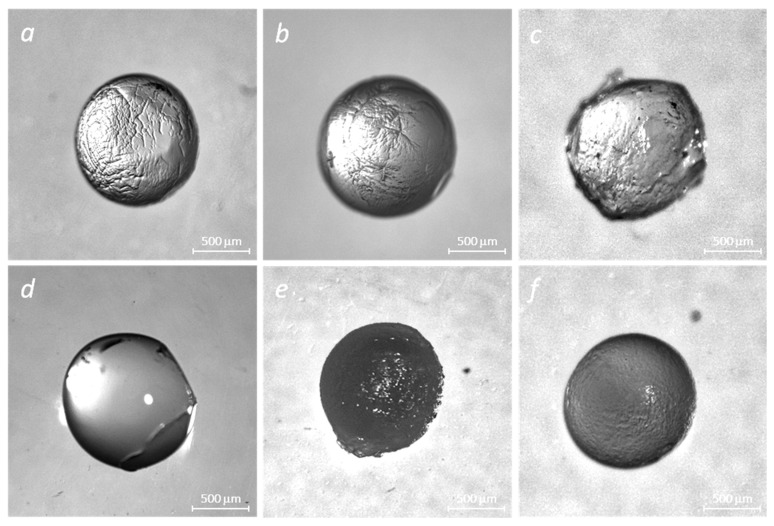
Stereo images of crosslinked polymer matrices of alginate and alginate–gelatin in the absence and presence of insulin: NaAlg + CaCl_2_ (**a**), NaAlg + Gel + CaCl_2_ (**b**), NaAlg + Gel + CaCl_2_ + GA (**c**), NaAlg + Gel + Ins (CaCl_2_) (**d**), NaAlg + Gel + Ins + CaCl_2_ + GA (**e**), and NaAlg + Ins + CaCl_2_ (**f**).

We used optical microscopy to observe the beads’ morphology changes for various formulations and designs. The results are depicted in Figure 4.

### 2.3. Scanning Electron Microscopy

All samples of hydrogel beads (alginate, alginate–gelatin) loaded with insulin, regardless of the type of crosslinking agents, have well-recognizable pores. Perhaps the formation of pores in the insulin-loaded samples (Figure 4) results from the insulin-matrix adsorption process.

A significant disruption of the hydrogel matrix observed in NaAlg-Insulin (CaCl_2_) samples may result in a poor delivery mechanism. Surface irregularities of the alginate surface are decreased with the addition of gelatin, suggesting an increase in matrix stability.

**Figure 5:**
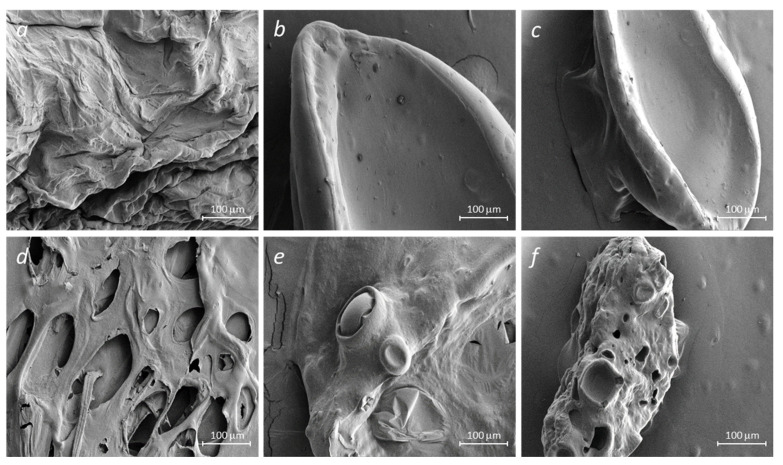
SEM images of crosslinked polymer matrices of sodium alginate and gelatin without (**a**–**c**) and with the embedded insulin (**d**–**f**): NaAlg + CaCl_2_ (**a**), NaAlg + Gel + CaCl_2_ (**b**), NaAlg + Gel + CaCl_2_ + GA (**c**), NaAlg + Ins + CaCl_2_ (**d**), NaAlg + Gel + Ins + CaCl_2_ (**e**), and NaAlg + Gel + Ins + CaCl_2_ + GA (**f**).

The morphology of the original and insulin-loaded alginate samples was examined using SEM (see Figure 5). Alginate particles loaded with insulin (Figure 5) (NaAlg-Insulin (CaCl_2_)) showed a porous structure compared to the initial sample. It is possible to notice the formation of a smooth surface when gelatin is added to sodium alginate since the surface of the initial alginate (Figure 5 NaAlg (CaCl_2_)) has a non-uniform structure. Receptacles of biconcave shape (Figure 5 NaAlg-Gel-Insulin (CaCl_2_) and Alg-Gel-Insulin (CaCl_2_-GA)), and hollow spaces (Figure 5 Alg-Gel-Insulin (CaCl_2_-GA)), were found in the SEM images obtained during the immobilization of insulin into the alginate–gelatin composition. Such changes in the morphology of the examined samples may also confirm the interaction of insulin with NaAlg and the NaAlg-Gel polymer composition. The non-homogenous matrix seen in NaAlg (CaCl_2_) samples can result in complications if used as a delivery system. For this reason, insulin is highly immobilized (or adsorbed) on the surface of alginate and gelatin. As a result, pores form on the alginate surface and alginate–gelatin films, which may indicate successful insulin immobilization.

The observed degree of matrix inhomogeneity (porosity) is as follows: NaAlg + Ins + CaCl_2_ (d) > NaAlg + Gel + Ins + CaCl_2_ + GA (f) > NaAlg + Gel + Ins + CaCl_2_ (e).

### 2.4. Swelling Behavior and Encapsulation Efficacy

Understanding the swelling behavior of encapsulating beads in gastric fluid is crucial for optimizing drug delivery systems. It provides insights into the beads’ response to physiological conditions, aiding in the design of controlled release formulations. Knowledge of swelling helps to tailor the encapsulation matrix to withstand the acidic and enzymatic environment of the stomach, enhancing the stability and performance of the insulin delivery system. In the gastric fluid environment, characterized by its acidic pH and the presence of digestive enzymes like pepsin, the crosslinked alginate beads exhibit distinctive swelling behavior presented in Table 1.

Particles fabricated with sodium alginate crosslinked with CaCl_2_ demonstrated the most extensive swelling and the lowest insulin encapsulation efficiency. We attribute it to the well-developed porous structure (see Figure 5d) that allows SGF access to the internal compartment and solubilize adsorbed insulin. Such a leakage results in relatively low encapsulation efficiency. The gelatin and pure glutaraldehyde system demonstrates the lowest degree of swelling and one of the lowest values of encapsulation efficiency. Rapid formation of the strong, rigid matrix with a high degree of crosslinking may result in insulin expulsion due to glutaraldehyde-induced shrinkage. The acidic conditions induce the protonation of amino acid residues in gelatin, leading to electrostatic repulsions and subsequent swelling as water is absorbed into the gelatin matrix. Simultaneously, the ionotropic gelation of sodium alginate with calcium ions contributes to the crosslinked structure of the beads [21]. Given its sensitivity to pH changes, incorporating sodium alginate introduces a pH-responsive component. This dual responsiveness to pH and enzymatic activity creates a unique swelling behavior in the mixed beads. The gelatin–alginate blends demonstrate dramatically decreased swelling. However, encapsulation efficiency is also determined by the type of crosslinking agent. As in the case of pure sodium alginate, the use of CaCl_2_ as a crosslinking agent in the blend results in the formation of an extensive pore network, leading to the low encapsulation efficiency of insulin.

### 2.5. FTIR Spectroscopy of the Encapsulation Matrices and Insulin Delivery System

FTIR spectroscopy was used to obtain information about the composition of the matrices. The obtained spectra were interpreted based on known vibrational frequencies of functional groups and peaks corresponding to specific alginate, gelatin, and insulin bonds.

**Figure 6:**
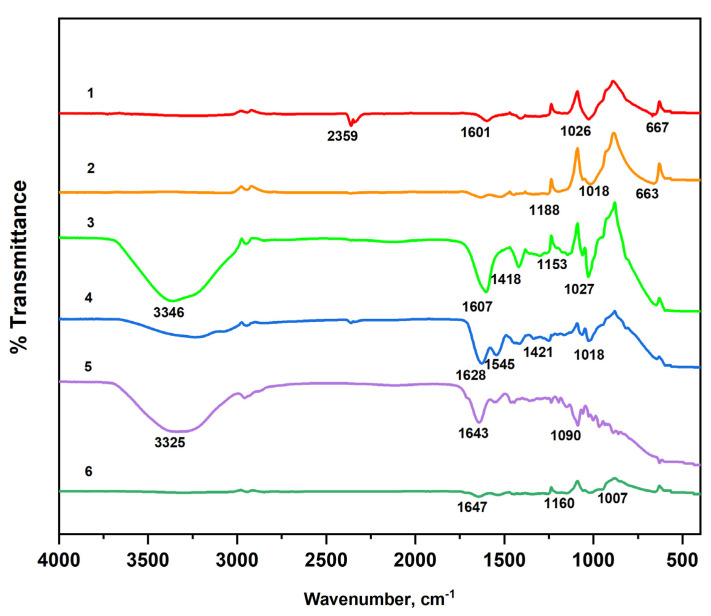
FTIR spectra of the raw materials (1—NaAlg; 2—Gel) and fabricated matrices (3—NaAlg (CaCl_2_), 4—NaAlg-Gel (CaCl_2_), 5—NaAlg-Gel (GA), and 6—NaAlg-Gel (CaCl_2_-GA)).

The FTIR spectra of the sodium alginate, gelatin, and fabricated systems using different crosslinkers are shown in Figure 6. Analysis of FTIR spectra of NaAlg and NaAlg-gelatin membranes shows that the carboxyl group band (1593 cm^−1^) in NaAlg shifted toward higher wavenumbers (1602, 1608, and 1622 cm^−1^ for NaAlg-gelatin (10), NaAlg-gelatin (30), and NaAlg-gelatin (50), respectively). Simultaneously, the intensities of bands at 1593 and 1411 cm^−1^ of pure NaAlg gradually decreased with increasing gelatin content. When comparing pure gelatin with the PEC membranes, the characteristic peaks of gelatin at 1637 cm^−1^ and 1554 cm^−1^ shifted to lower wave numbers. These changes suggest the formation of strong intermolecular interactions, including hydrogen bonding and electrostatic attractions between NaAlg and gelatin chains. The incorporation of gelatin altered the loose packing of NaAlg polymer chains, resulting in higher packing efficiency and a more regular structure [22,23]. In the FTIR spectrum of the NaAlg matrix, characteristic absorption bands at 1593 and 1411 cm^−1^ corresponded to –COO^−^ asymmetric and symmetric stretching peaks, respectively [24]. Additional bands or shoulders around 1310 (C–O stretching), 1088 (C–O stretching), 1031 (CO–C stretching), and 946 cm^−1^ (C–O stretching) were attributed to its saccharide structure [25]. The gelatin spectrum exhibited strong bands at 1637 cm^−1^ and 1554 cm^−1^, associated with amide carbonyl (C–O and C–N stretching vibration) and bending vibration of –NH. Bands ranging from 1225 cm^−1^ to 1040 cm^−1^ represent characteristic peaks of amino and alkyl chains [26,27].

**Figure 7:**
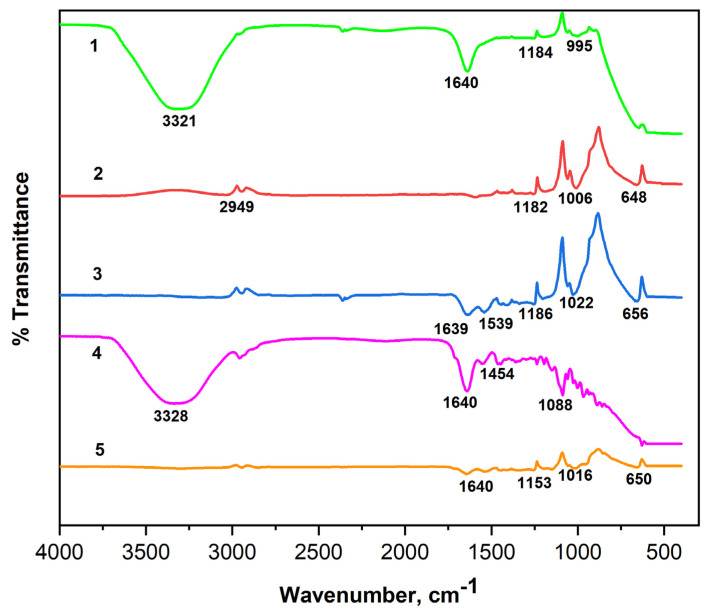
FTIR spectra of the insulin (1) and insulin-loaded hydrogel matrices: 1—Insulin; 2—NaAlg-Insulin (CaCl_2_); 3—NaAlg-Gel-Insulin (CaCl_2_); 4—NaAlg-Gel-Insulin (GA); 5—NaAlg-Gel-Insulin (CaCl_2_-GA).

The FTIR spectra of the NaAlg and NaAlg-Gel systems loaded with insulin using different crosslinkers are shown in Figure 7. The 3500–3000 cm^−1^ peaks correspond to the polymer matrices’ hydroxyl groups (–OH) [28]. The absorption band at a wavelength of 2949 cm^−1^ is a characteristic peak of C–H stretching vibrations and is present in alginate particles loaded with insulin [29]. Intense peaks in the range of 1643–1599 cm^−1^ in the samples of NaAlg-Gel composition correspond to N-H bending vibrations and N–C=O stretching vibrations of amide II [30] and prove the presence of a peptide bond in NaAlg-Gel composition. Bands characterizing C-C stretching of aromatic compounds were found in the 1418–1454 cm^−1^ range. Vibration bands at 1006–1300 cm^−1^ refer to C–O and C–H deformations, also found in all the given spectra [31]. In the samples loaded with insulin in the 667–648 cm^−1^ range, new peaks of deformation vibrations of O=N–O bonds were detected. It should be noted that the peak of 1640 cm^−1^ registered in the insulin spectrum, despite differences in the intensity of the absorption bands, remained unchanged in all spectra of the NaAlg-Gel composition loaded with insulin. The constant position of this peak may indicate the stability of immobilized insulin. When comparing the presence of insulin in NaAlg (CaCl_2_), a significant loss of peaks is noted between 1600 and 1000 cm^−1^. However, with the addition of gelatin, many of these peaks are preserved, confirming effective insulin encapsulation in gelatin-containing matrices.

### 2.6. Molecular Dynamics Simulations

Potential energy is a measure of the total energy associated with the relative positions of atoms in a molecule. We used Molecular Mechanics 2 (ChemOffice Ultra, PerkinElmer, Waltham, MA, USA) to model the potential energy of designed polymeric matrices to reveal the mechanism of intermolecular interactions. In MM2, the potential energy of a molecular system is computed as a sum of the energy terms associated with specific atomic interactions, such as bond stretching energy, angle bending energy, torsional (dihedral) energy, van der Waals energy, and electrostatic energy (see Table 2). Since gelatin is a complex mixture of many amino acids, we use glycine and proline, the major constituents of the gelatin. [32] in calculations of the interaction energies of gelatin. All molecular structures were created and analyzed in ChemDraw Professional Suite 22.

According to the simulation results, dipole–dipole interactions are predominant in the insulin encapsulation process: NaAlg-Insulin (81%), Gel-Insulin (97%), and Ins-Ins (88%). They account for over 80% of the total interaction energy, confirming that electrostatic attraction is the most dominating mechanism in the developed matrices. Gelatin–alginate interactions are mostly van der Waals-type with insufficient contribution from the polar energy component. The relatively weak electrostatic potential between gelatin and alginate indicates a hybrid gel structure when all constituents are independently crosslinked, forming interpenetrating gel networks [33].

## 3. Discussion

There are three aspects in the evaluation of the comparative overall efficiency of the designed hydrogel particles for potential insulin delivery applications: the swelling behavior of the particles in the stomach environment, characterizing the ability of the matrix to prevent insulin degradation; encapsulation efficiency characterized by the hydrogel ability to hold/immobilize insulin; and, finally, the ability of the designed matrix effectively encapsulated insulin in the most bioavailable form preventing its self-assembly and fibrils formation.

The swelling behavior of crosslinked gelatin and alginate beads and the complex interaction between these two biopolymers in gastric fluid is critical for designing such a hybrid hydrogel delivery system. Alginate gels demonstrate a pH-dependent behavior in the gastric environment. The acidic pH can trigger alterations in the alginate matrix, leading to increased water uptake and subsequent swelling. One of the primary mechanisms contributing to the swelling is ion exchange. In the stomach’s acidic environment, protons (H⁺ ions) replace the calcium ions within the alginate matrix. This ion exchange disrupts the crosslinked structure, leading to repulsive forces between alginate chains. Consequently, the beads swell as they absorb water and expand in response to the altered ionic interactions. Additionally, the presence of pepsin in gastric fluid contributes to the degradation of the alginate matrix, inducing erosion and breakdown of the alginate structure. On the other hand, the acidic conditions of the stomach induce the protonation of amino acid residues in gelatin, leading to electrostatic repulsions and consequent swelling as water is absorbed into the gelatin matrix. Additionally, pepsin’s enzymatic activity contributes to gelatin’s degradation, causing changes in the structural integrity of the beads and influencing their swelling characteristics. The pH sensitivity of gelatin, combined with its susceptibility to enzymatic degradation, affects the overall swelling behavior of the beads in gastric fluid.

Calcium ions play a critical role in crosslinking alginate molecular chains. In the presence of insulin, calcium ions can be exchanged between the alginate matrix and the insulin molecules. Insulin may competitively bind to the alginate, displacing calcium ions and disrupting the crosslinked structure. As the matrix swells in the gastric environment, the spaces between the polymer chains increase, allowing insulin molecules to diffuse out of the matrix. Particles prepared from an alginate blend and crosslinked in the presence of calcium and glutaraldehyde exhibit moderate swelling resistance and encapsulation efficiency. The expulsion/leakage of insulin from an alginate matrix crosslinked with calcium involves several potential mechanisms, reflecting the dynamic interaction between the matrix and the encapsulated insulin.

The crosslinking process with glutaraldehyde increases the density of the gelatin matrix. The degree of crosslinking influences the matrix’s permeability and insulin migration. As determined by the swelling coefficient data, a strong crosslinking leads to a more rigid matrix, affecting insulin encapsulation efficiency. We hypothesize that insulin was expelled from the matrix into the crosslinking bath during simultaneous shrinkage and pore formation in the hydrogel matrix.

**Figure 8:**
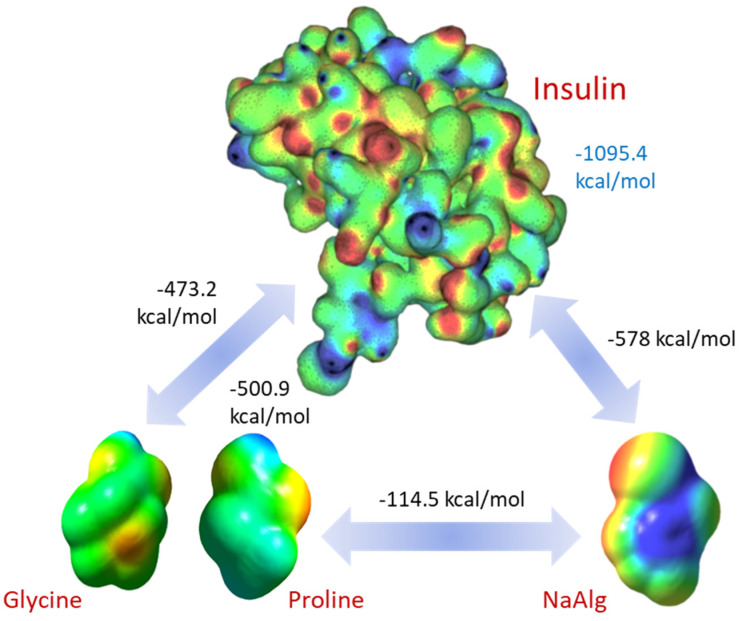
Computed electrostatic potential maps of the interacting molecules and pairwise total interaction energies. The electrostatic potential map for insulin has been adapted from [34].

Molecular modeling provided more insight into the interaction between the hydrogel components and insulin. The electrostatic potential map shows regions with strong and weak electrostatic potential in the molecules. Regions colored in red indicate a strong electron-attracting potential attributed to the existence of electronegative groups. In contrast, blue-colored regions indicate a low-level electron-donating potential. Figure 8 depicts the optimized structures of insulin, sodium alginate, and gelatin amino acids alongside their corresponding electrostatic potential map.

Insulin–insulin interaction has the highest energy of 1095.4 kcal/mol, which explains its tendency for self-assembling and fibril formation well. Surprisingly, both gelatin (glycine, 473.2 kcal/mol, proline, 500.9 kcal/mol) and sodium alginate (578 kcal/mol) show strong, attractive electrostatic potentials towards insulin, providing solid support for the proposed mechanism of the insulin encapsulation. This also supports our finding that interaction with the excipient molecules impacts fibril formation. Despite the lower value of the potential interaction, insulin within the encapsulation matrices has a much lower concentration. Therefore, the number of interaction sites greatly exceeds the number of such sites on the insulin molecules. As a result, we can expect reduced fibril formation due to competitive electrostatic adsorption between insulin, alginate, and gelatin.

**Figure 9:**
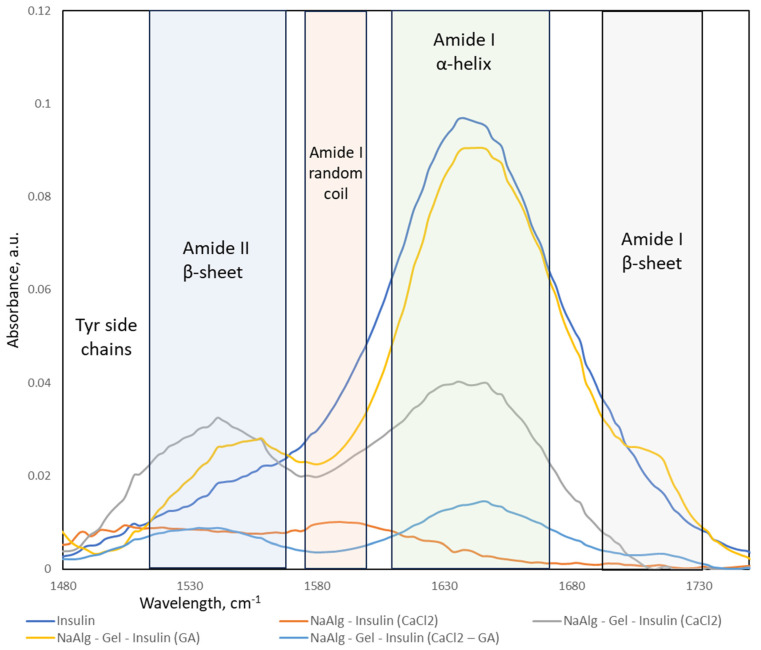
FTIR spectra in 1500–1700 cm^−1^ region and corresponding band assignment of insulin.

The FTIR spectra (Figure 9) of sodium alginate (NaAlg), gelatin, and NaAlg–gelatin blends with encapsulated insulin in the 1700–1500 cm^−1^ range are the most important for understanding conformational changes in the system. Insulin spectra show strong peaks associated with the Amyloid I α-helix structure, a small fraction of Amyloid I β-sheets, and shoulder bands in the Amyloid II region. FTIR spectra of insulin-loaded hydrogel particles exhibit strong blue-shifted spectra, as demonstrated in [35]. A strong blue shift is typically associated with changes in the molecular environment or interactions within a matrix. One common cause of blue shifts in IR bands is the formation of hydrogen bonds. Hydrogen bonding can alter the vibrational modes of molecules, leading to shifts in the absorption bands. As confirmed by the molecular modeling results, the hydrogen bonds are the primary mechanism of the insulin association with the alginate and gelatin.

Insulin, a crucial hormone in regulating blood sugar, has been found to form amyloid fibrils under certain conditions. Under conditions that promote aggregation, insulin molecules can undergo conformational changes and assemble into fibrillar structures characterized by beta-sheet formations. This aggregation process is associated with the conformational misfolding of insulin molecules, forming insoluble aggregates. The process of fibril formation can be controlled by altering the interaction of insulin with the surrounding environment (sodium alginate and gelatin) and influencing the conformational changes in the protein.

Insulin amyloid fibrils are composed of misfolded proteins or peptides that aggregate into insoluble fibrils with a characteristic cross-β-sheet structure. The presence of β-sheet structures is typical for the fibrils [36]. The formation of β-sheets in insulin fibrils indicates an increase in the structural organization of the protein. FTIR spectra collected from the insulin-loaded hydrogel particles were processed (baseline removal and deconvolution). The calculated fractional area of the peaks represents the relative composition of the insulin secondary structures in the fabricated particles (see Table 3).

Original insulin formulation contains mostly Amyloid I α-helix, the most bioavailable insulin configuration. A minor fraction of Amyloid II β-sheet indicates weak aggregation due to high insulin concentration and long storage time. Sodium alginate particles crosslinked via ionotropic gelation contain mostly Amyloid I random coils and some tyrosin chains. The insulin signal was very low, which agrees with our encapsulation efficiency data. No fibril formation is observed in NaAlg-Insulin (CaCl_2_) formulation.

All formulations containing gelatin show a strong presence of Amyloid I α-helix, with the highest fractions in those that were crosslinked with glutaraldehyde. However, blended hydrogels that were crosslinked in the presence of the CaCl_2_ contain a lower fraction of the Amyloid I α-helix and significantly higher content of the Amyloid II β-sheets. It strongly indicates the fibril formation process, making such formulations less preferable for drug delivery applications. There is a small fraction of Amyloid I β-sheet conformation that might be a result of insulin interaction with glutaraldehyde.

Fibril formation involves the transition of protein structures from random coil or α-helical conformations to β-sheet-rich structures. As observed, blends with the gelatin prevent fibril formation and leakage of the insulin from the hydrogel. We speculate that the prevention of fibril formation through the interaction with amino acids can be attributed to competitive adsorption between gelatin chains and insulin molecules.

The most common mechanisms through which amino acids or other agents can prevent fibril formation are disruption of nucleation via hindrance/steric factor, inhibition of β-sheet formation, and diffusion transport limitation. Gelatin amino acids, especially those with hydrophobic or aromatic side chains, can competitively occupy binding sites on the protein surface critical for intermolecular interactions leading to fibril formation. Amino acids with hydrophobic side chains can interfere with hydrophobic interactions that drive protein aggregation. By competitively binding to hydrophobic regions on the protein surface, they disrupt the hydrophobic forces contributing to fibrillogenesis.

Additionally, amino acids can introduce steric hindrance by physically blocking the interaction sites on the insulin molecules. This inhibits the close proximity required for the formation of stable nuclei [37]. If a nucleus is formed, amino acids that disrupt the formation of β-sheet structures, characteristic of fibrils, can competitively bind to the regions where these interactions occur. This disrupts the stacking of β-sheets and hinders the growth of fibrils. Amino acids with charged side chains can influence electrostatic interactions involved in protein aggregation. By competitively binding to charged regions on the insulin molecule, they impede the progression of fibril formation. Additionally, large biopolymeric molecules provide significant diffusion resistance, often called “molecular crowding”, that influences fibril formation by increasing the time for protein aggregation.

## 4. Materials and Methods

### 4.1. Materials

All materials were of chemical grade and stored according to the manufacturer’s specifications. Sodium alginate (Alg), viscosity 25 cps at 1%, pH = 6.3 (Fit Lane Nutrition, Sacramento, CA, USA), gelatin (Gel), Bloom index = 241, pH 5.3 (The Kraft Heinz Company, Chicago, IL, USA), calcium chloride (Fisher Scientific Company, Waltham, MA, USA), glutaraldehyde (GA), (Sigma-Aldrich Co., Darmstadt, Germany), insulin isophane (human, genetically engineered, 32 UI, Novo Nordisk A/S, Bagsværd, Denmark).

### 4.2. Methods

#### 4.2.1. Bead Preparation: Ionotropic Gelation and Crosslinking

Systems consisting of sodium alginate and sodium alginate–gelatin were chosen as hydrogel-forming matrices for insulin encapsulation. Two crosslinking agents, CaCl_2_ and GA, and their combination CaCl_2_-GA, will be used for gelation.

Alginate beads were fabricated using a standard ionotropic gelation method. A 1% (*w*/*v*) sodium alginate solution was prepared by dissolving sodium alginate in distilled water. This solution was then extruded with a syringe pump (New Era Pump Systems NE-300, Inc., Temecula, CA, USA) into a crosslinking bath solution, forming stable hydrogel beads. In the case of sodium alginate, the crosslinking agents were 1.5% CaCl_2_ and 1.5% CaCl_2_-25% GA. NaAlg-Gel matrices were obtained by mixing 1% NaAlg solution and 8% Gel solution in a ratio of 1:1 (V_1_:V_2_) and then adding the resulting mixture using a syringe pump into a solution of crosslinking agents; in the case of the NaAlg-Gel system, the crosslinking agents were 1.5% CaCl_2_; 25% GA; 1.5% CaCl_2_; and 25% GA. Obtained hydrogel particles were kept in solutions of crosslinking agents for 30 min, collected by filtration, and washed with deionized water to remove excess calcium chloride, filtered, and dried under ambient conditions.

To immobilize insulin into the obtained polymer matrices, 50 mL of 1% NaAlg solution and 50 mL of a mixture of NaAlg-Gel containing 0.28 mg/mL of insulin was added dropwise using a syringe pump into solutions of crosslinking agents. Crosslinkers for NaAlg were CaCl_2_ and CaCl_2_-GA. Crosslinkers for NaAlg-Gel were CaCl_2_, GA, and CaCl_2_-GA. After 30 min, the formed particles were collected by filtration and washed with deionized water.

#### 4.2.2. Optical Microscopy

The shape and surface morphology of the beads were characterized by optical microscopy using an SMZ-171 TLED stereomicroscope (Motic Asia, Hong Kong, China) equipped with the Moticam Pro S5 Plus RMP digital microscope camera. For the analysis, fabricated particles were gently dried with the filter paper and placed onto a microscope slide. The diameter of the beads was determined using an optical microscope and digital micrometer. All images were acquired using MoticImage Plus 2.0 and processed with ImageJ software (Version 15.4i, NIH, Bethesda, ML, USA).

#### 4.2.3. Scanning Electron Microscopy

The initial and insulin-loaded samples were cut into two parts using a straight-bladed laboratory micro-razor, securely placed to double-sided conductive carbon tabs (Ted Pella, Redding, CA, USA), and dried at room temperature under vacuum. After that, the samples were coated with a thin layer of gold using EMS 150T ES Coater (Quorum Technologies, Lewis, UK). FE-SEM images were taken with a SIGMA FE-SEM field emission scanning electron microscope (ZEISS Sigma, Jena, Germany). SEM images were acquired with E = 5 kV and a working distance of 8.1–9.4 mm.

#### 4.2.4. FTIR Spectroscopy

Before analysis, the fabricated beads were securely placed on the ATR crystal of the Thermo Nicolet IR Fourier spectrometer (Thermo Electron Corporation, Waltham, MA, USA). A background spectrum was recorded using an empty ATR crystal for baseline correction. Subsequently, multiple scans (e.g., 64 scans) of the alginate beads were collected in the mid-infrared region (4000–400 cm⁻^1^). The acquired FTIR spectra were analyzed for characteristic peaks corresponding to functional groups present in alginate. Peaks associated with the O–H stretching vibration, C–H stretching vibration, and C=O stretching vibration were examined. The spectra were baseline-corrected, and the peak positions and intensities were determined.

Control experiments were conducted using fabricated beads without encapsulated insulin to confirm the specificity of the observed peaks in the obtained spectra.

#### 4.2.5. Determination of the Degree of Swelling

The swelling behavior of alginate beads in the gastric fluid was assessed following a standard experimental protocol with some modifications [38]. Simulated Gastric Fluid (SGF) was prepared to mimic the physiological conditions of the stomach. The SGF composition included 0.1 N hydrochloric acid (HCl) to replicate an acidic (pH 1.2) gastric environment, and pepsin was added to simulate the presence of digestive enzymes.

The swelling after 30 min of the beads–SGF contact time has been determined to evaluate the encapsulation matrix resistance to the stomach conditions. A predetermined number of alginate beads were carefully submerged in a specified volume (4.5 mL) of SGF to initiate the swelling experiments. The beads swelled under controlled conditions at 37 °C (VWR digital water bath, filled with LabArmor thermal beads). After 30 min, bead samples were removed from the SGF medium, and excessive gastric fluid was removed with filter paper to absorb excess liquid from the surface. The degree of swelling (swelling index) was determined after 30 min of direct contact of hydrogel beads with the gastric environment. The mass of the beads was determined before and after the SGF experiment with the analytical weigh module WXSS205 (Mettler-Toledo, Columbus, OH, USA). The degree of swelling was quantified by calculating the swelling index (*S*), which represents the ratio of the swollen bead weight to the initial bead weight $S=\left(m_{t}-m_{0}\right)/m_{0}$, where $m_{t}$ and $m_{0}$ are the initial and final sample weights.

Control experiments were conducted using fabricated beads immersed in neutral solutions to differentiate the specific effects of the gastric environment on swelling behavior.

#### 4.2.6. Determination of Drug Content and Encapsulation Efficiency

The insulin concentration was determined by measuring the absorption of phosphate buffer at 275 nm. The measurements were performed using a high-resolution UV-VIS-NIR spectrometer HR2000+ (Ocean Insights, Orlando, FL, USA) and a deep UV 275 nm LED M275L4 (Thorlabs, Newark, NJ, USA) as the light source. The encapsulation efficacy of insulin in the fabricated hydrogel beads was determined as follows: cross-linked beads were removed from the curing bath and gently wiped with filter paper to remove the excess crosslinking agent. Ten beads loaded with insulin were placed in a standard spectrophotometric quartz cuvette (Hellma GmbH, Müllheim, Germany) with a 5 mL internal volume filled with a sodium phosphate buffer (pH = 7.4), covered with a Teflon cap, placed onto a heated magnetic stirrer set to 37 °C, and incubated for 24 h. After incubation, the cuvette was transferred to the spectrophotometer, and absorption at 275 nm was measured. The total quantity of drug released over 24 h was measured spectrophotometrically and interpreted as the total amount of encapsulated insulin. Insulin encapsulation efficiency was evaluated by calculating the ratio of the amount of insulin encapsulated (based on formulation) to the overall amount of insulin released into the dissolution media [38].

#### 4.2.7. Molecular Dynamic Simulation Methodology

The Molecular Mechanics 2 (MM2) modeling software (ChemOffice Ultra, PerkinElmer, Waltham, MA, USA) was used for potential energy modeling of hydrocolloid matrices to investigate intermolecular interactions. The process involved constructing atomistic models of the molecules, i.e., the creation of the .mol files, MM2 calculations including bond stretching, angle bending, torsional strains, and non-bonded interactions (van der Waals and electrostatic forces). We systematically analyzed pair-wise interactions of the encapsulation matrix components with embedded insulin molecules. This approach enabled us to quantitatively assess the influence of molecular structural changes on the matrices’ properties, which is crucial for understanding insulin conformation mechanisms in the designed delivery system.

## 5. Conclusions

This study investigated the effect of the hydrogel matrix composition and type of crosslinked agent. For the immobilization of insulin, systems consisting of polymer matrices alginate and alginate–gelatin crosslinked with the following crosslinking agents: CaCl_2_, GA; CaCl_2_-GA. Two hydrogel components have different pH sensitivity and different mechanisms of gel formation. Using a variable gelling agent, we produced various structures that greatly differed in surface morphology, internal structure, and swelling behavior. Optical and field emission scanning electron microscopy were used to study the structure and morphology of insulin-loaded beads. Observed well-developed porous structure in alginate matrices attributed to the alginate–insulin interactions leads to insulin leakage and low encapsulation efficiency. FTIR spectroscopy was used to analyze intermolecular interactions. Absorption maxima in the range of 1643–1599 cm^−1^ prove the presence of a peptide bond in the alginate–gelatin composition. In all spectra of the NaAlg-Gel composition loaded with insulin, the peak registered in the spectrum of insulin at 1640 cm^−1^ remained unchanged, along with the improved intensity of other peaks between 1600 and 1000 cm^−1^, which may indicate the stability of immobilized insulin. In the SEM images obtained during the immobilization of insulin in the NaAlg-Gel composition, biconcave receptacles and voids were found, which may be the result of the interaction of insulin with the gelatin. Hydrogel beads produced with glutaraldehyde crosslinked alginate–gelatin blends demonstrated superior encapsulation, efficiency, and resistance to the simulated stomach environment. It was found that gelatin prevents β-sheet formation in insulin, slowing down the process of fibrilization.

The MM2 simulation results provided a strong computational validation for our experimental observations. Specifically, the simulations were in complete agreement with the FTIR data about the hydrogel matrix composition and its interaction with insulin. The MM2 model effectively demonstrated how different matrix compositions and crosslinking agents influenced the structural integrity and stability of insulin within the hydrogel matrices. The MM2 simulations enabled a deeper understanding of the molecular interactions within these matrices, offering insights into the mechanism of the observed insulin leakage and encapsulation efficiency. The obtained results provided a strong theoretical foundation for the understanding of the insulin immobilization mechanism in different hydrogel compositions.

Obtained data confirm that manipulating the microenvironment around insulin makes it possible to design delivery systems that disrupt the insulin aggregation process, preventing the undesired formation of amyloid fibrils. A demonstrated approach to designing hybrid hydrogel matrices enables the tailoring of the swelling behavior and potentially optimizes controlled release profiles for encapsulated drugs in response to the specific physiological conditions of the gastrointestinal tract.

## Figures and Tables

**Table 1:** Swelling behavior and encapsulation efficacy of the insulin delivery systems.

System	Encapsulation Efficiency, PBS	Swelling Coefficient, SGF, 10 min	Swelling Coefficient, SGF, 30 min	Observation
NaAlg + CaCl_2_ + Ins	67.4 ± 2.4	1.6 ± 0.18	2.4 ± 0.23	Leakage
Gel-GA + Ins	70.2 ± 3.3	1.1 ± 0.16	1.2 ± 0.18	Expulsion
NaAlg + Gel + CaCl_2_ + Ins	74.1 ± 1.8	1.2 ± 0.11	1.4 ± 0.12	Good
NaAlg + Gel + GA + Ins	81.2 ± 2.3	1.1 ± 0.09	1.3 ± 0.14	Good
NaAlg + Gel + CaCl_2_ + GA + Ins	72.4 ± 2.1	1.3 ± 0.12	1.8 ± 0.21	Leakage

**Table 2:** Computed intermolecular interaction energies in the Alg-Gel-Insulin system.

Energy (kcal/mol)	Sodium Alginate	Proline	Glycine	Insulin
Sodium Alginate	−215.5	−81.9	−114.5	−578.1
Proline	−81.91	11.19	0.757	−501.0
Glycine	−114.47	0.76	−17.31	−473.2
Insulin	−578.1	−501.0	−473.2	−1095.4

**Table 3:** Fractional analysis of the insulin conformations (%) in fabricated hydrogel particles.

Formulation	Amyloid I a-Helix	Amyloid I b-Sheet	Amyloid I Random	Amyloid II b-Sheet	Tyr Side Chains
Insulin	89	-	-	11	-
NaAlg-Insulin (CaCl_2_)	-	-	54	-	46
NaAlg-Gel-Insulin (CaCl_2_)	55	-	-	45	-
NaAlg-Gel-Insulin (GA)	75	7	-	18	-
NaAlg-Gel-Insulin (CaCl_2_-GA)	59	4	-	37	-

## Data Availability

Data are contained within the article.
